# Mitophagy and reactive oxygen species interplay in Parkinson’s disease

**DOI:** 10.1038/s41531-022-00402-y

**Published:** 2022-10-18

**Authors:** Bin Xiao, Joshua Kuruvilla, Eng-King Tan

**Affiliations:** 1 Department of Neurology, National Neuroscience Institute, Singapore, Singapore; 2 Neuroscience Academic Clinical Program, Duke-NUS Medical School, Singapore, Singapore; 3 Neuroscience and Behavioral Disorders Program, Duke-NUS Medical School, Singapore, Singapore

**Keywords:** Medical research, Molecular biology

## Abstract

Mitophagy impairment and oxidative stress are cardinal pathological hallmarks in Parkinson’s disease (PD), a common age-related neurodegenerative condition. The specific interactions between mitophagy and reactive oxygen species (ROS) have attracted considerable attention even though their exact interplay in PD has not been fully elucidated. We highlight the interactions between ROS and mitophagy, with a focus on the signalling pathways downstream to ROS that triggers mitophagy and draw attention to potential therapeutic compounds that target these pathways in both experimental and clinical models. Identifying a combination of ROS inhibitors and mitophagy activators to provide a physiologic balance in this complex signalling pathways may lead to a more optimal outcome. Deciphering the exact temporal relationship between mitophagy and oxidative stress and their triggers early in the course of neurodegeneration can unravel mechanistic clues that potentially lead to the development of compounds for clinical drug trials focusing on prodromic PD or at-risk individuals.

## Introduction

Parkinson’s disease (PD) is a progressive neurodegenerative condition identified as one of the leading causes of disability, particularly in countries with rapidly growing aging populations and with a prevalence that is predicted to double in the next 20 years^1–3^. A selective loss of dopaminergic (DA) neurons from the pars compacta of the substantia nigra (SN) and the formation of Lewy bodies constitute the primary pathological hallmarks of PD. Clinically, patients frequently present with tremor, rigidity, postural instability and bradykinesia accompanied by nonmotor symptoms (such as fatigue, depression, and dementia)^1^. The etiology of PD has been extensively investigated and is likely due to a combination of genetic, epigenetic and environmental factors. Clinical, postmortem and animal model studies suggest that mitochondrial dysfunction, oxidative stress, impaired proteasomal system, impaired autophagy/mitophagy, and neuroinflammation dysregulation^1,4,5^ contribute to the onset and development of PD. Available therapies are largely symptomatic, and disease-modifying therapies are still lacking, highlighting the necessity of therapy targeting key areas of PD pathogenesis^6^. There is growing evidence implicating that mitophagy impairment^7,8^ and oxidative stress^9,10^ are critical factors of neurodegeneration over time in PD.

### Mitophagy

Mitophagy refers to autophagy that selectively degrades damaged mitochondria. There exists a multi-tiered, highly integrated network of mitochondrial quality control (MQC) which regulates various cellular processes to maintain the structural and functional integrity of mitochondria in response to various stressors (oxidative stress, mutagenic stress, and proteotoxicity etc). Various MQC pathways are dysregulated in PD, including mitochondrial regeneration (biogenesis^11^, protein import^12^), mitochondrial dynamics (fission, fusion)^13,14^ mitochondria-derived vesicles^15^ and mitochondrial removal (mitophagy)^7,16^.

An essential component of mitochondrial homeostasis, mitophagy can be defined as the selective degradation of mitochondria, with mitochondria enveloped by autophagosomes before being degraded in the lysosome^17,18^. Given that this breakdown of mitochondria via autophagosome formation is crucial for neuronal health, compromised mitophagy has been implied in PD, and associated with accelerated neurodegeneration^19,20^. Based on their relevance to PD pathogenesis, mitophagy pathways can be categorised into Parkin-dependent and Parkin-independent pathways, with the latter largely comprising of receptor-mediated mitophagy, lipid-mediated mitophagy and ubiquitin-mediated mitophagy.

#### Parkin/PINK1-mediated mitophagy

##### Mechanisms of Parkin/PINK1-mediated mitophagy

Mutations in PINK1 (a serine/threonine kinase)^21^ and Parkin (an E3 ubiquitin ligase)^22^ are the most common causes of recessive forms of PD. PINK1 and Parkin are the key components in the Parkin/PINK1-mediated mitophagy^23,24^. Under basal conditions, PINK1 translocates to the inner mitochondrial membrane (IMM) for cleavage by mitochondrial proteases^25^. Subsequently, truncated PINK1 is targeted to the proteasome for eventual degradation via an N-end rule pathway^26^. Mitochondrial stress or damage causes the collapse of mitochondrial membrane potential, preventing this manner of translocation of PINK1 which instead accumulates on the outer mitochondrial membrane (OMM)^25,27^ and undergoes autophosphorylation^28^. In this initiation stage, PINK1 phosphorylates OMM conjugated ubiquitins at Serine 65, driving Parkin to be recruited from cytosol to the mitochondria^29,30^. Phosphorylated ubiquitin may both activate Parkin and act as an anchor for Parkin on mitochondria where Parkin is additively activated by PINK1 phosphorylation^24,29,30^.

The phosphorylation of ubiquitin and Parkin triggers a series of structural remodelling for Parkin, converting it from a dormant enzyme with self-inhibited conformation to an active and promiscuous E3 ligase which mediates ubiquitination of OMM proteins non-selectively^29,31–33^. Among the Parkin substrates, Miro were ubiquitinated and degraded to arrest mitochondrial motility, priming mitochondria for autophagic clearance^34^. Ubiquitin attached to the substrates on the damaged mitochondria can be phosphorylated by PINK1 to recruit and activate more Parkin and form a positive feedback loop to propel the cascade of mitophagy^29,31,35^. Subsequently, the ubiquitinated substrates serve as a degradation signal to be recognized by autophagy receptors, including OPTN and NDP52^23^. This occurs concomitantly with Parkin/PINK1-dependent activation of TBK1 which phosphorylates and recruits OPTN and NDP52 to the depolarized mitochondria, achoring them to the forming autophagosome coated with LC3^23,36,37^. The process is achieved via the development of an isolation membrane surrounding the mitochondria via an ATG8-dependent positive feedback loop^37^.

**Fig. 1:**
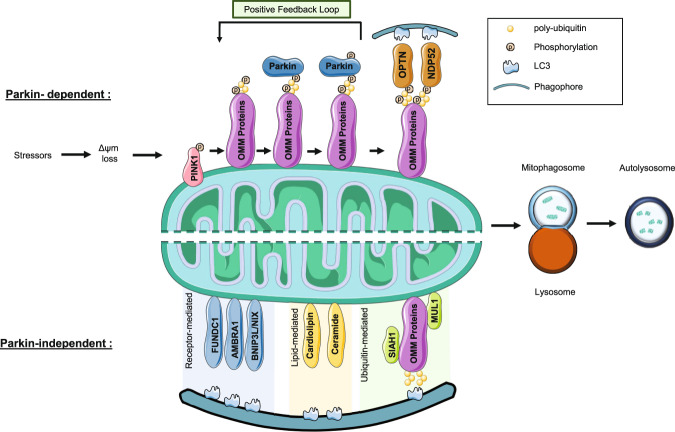
Primary mechanisms of Parkin-dependent and Parkin-independent mitophagy. In Parkin-dependent mitophagy, PINK1 is stabilized to the OMM and autophosphorylated following mitochondrial depolarization. This is followed by the phosphorylation of ubiquitin and Parkin, with Parkin being recruited towards OMM and activated, leading to the broad ubiquitination of OMM substrates. Mitophagy is executed via autophagy receptor proteins (OPTN, NPD52) which link the impaired mitochondria to autophagosome. In Parkin-independent mitophagy, this linking of mitochondria to autophagosome is achieved in the absence of Parkin, with three pathways and their key players, including but not limited to: receptor-mediated mitophagy (FUNDC1, AMBRA1, BNIP3/NIX), lipid-mediated mitophagy (Cardiolipin, Ceramide), and ubiquitin-mediated mitophagy (SIAH1, MUL1).

The damaged mitochondria are engulfed by the autophagosome which subsequently fuses with lysosome containing acidic hydrolases^37–39^. Mitophagy is completed as the autophagosome-lysosome fusion leads to the degradation of mitochondria^38,40^ (Fig. 1). In addition, several Parkin-independent mitophagy pathways have been identified, which will be discussed later, having the potential to be utilized to compensate for the dysfunction of Parkin-mediated mitophagy.

##### Genetic and pathological evidence of Parkin/PINK1-mediated mitophagy

Parkin and PINK1 mutations disrupt their physiologic functions on mitophagy. Extensive studies in cultured cells have not only unravelled the molecular mechanism underlying Parkin/PINK1 pathway discussed above, but also revealed that over half of tested PD-related mutations in Parkin and PINK1 are associated with defect in mitophagy^27,41–43^. Furthermore, there are in vivo evidence showing that mitophagy dysfunction impacts DA neuronal health and contributes to PD pathogenesis. Phosphorylation of ubiquitin at Ser65, a hallmark of PINK1 activation during mitophagy was increased in a Mutator mouse with mitochondrial dysfunction, and loss of endogenous Parkin leads to specific DA neurodegeneration and L-DOPA reversible motor deficit^44^. Studies using Drosophila model expressing mitophagy probes demonstrated Parkin/PINK1-dependent mitophagy activation in DA neurons in an age-dependent manner^45^. Another study demonstrated that depletion of deubiquitinase UPS30 in fly may rescue Parkin/PINK1-dependent mitophagy and counteract PD-like symptoms of paraquat-induced decreased dopamine level and motor dysfunction^46^. In addition to Parkin and PINK1 which play a fundamental role in mitophagy, other PD causative genes, including α-synuclein, LRRK2 and GBA, may contribute to PD pathogenesis also through their impact on mitophagy which will be discussed below.

Abnormality in PINK1-dependent mitophagy induced by aging can be inferred with the accumulation of PINK1 activity biomarker, pS65-Ub found in elderly human subjects^47^. Further pathological evidence of altered mitophagy was found in aging PD brain samples where there were increased mitochondrial matrix protein, ATP synthase subunit β (ATP5β), and OMM protein, Miro. Upregulation of Miro may increase mitochondrial motility and compromise the initiation of mitophagy. Therefore, aberrant accumulation of ATP5β and Miro in postmortem brain indicates that dysfunctional mitophagy is associated with PD^16^. PD patient platelets, compared to aged-matched controls, exhibited downregulated mitophagy accompanied by reduced LC3II and MsrB2, which affects Parkin methionine oxidation^48^. These pathological and clinical findings are indicative of defective mitophagy in PD.

##### Evidence of Parkin/PINK1-mediated mitophagy from patient iPSC-derived neurons

Although mutations in Parkin or PINK1 were found to compromise mitophagy in cultured cells and animal models, direct pathological evidence is still lacking if the same mutations would impair mitophagy and if such defect is the key factor for the loss of DA neurons in vivo or in PD patients^49^. Recent advancements in stem cell and gene editing technology may help address these questions. The impact of disease-causing mutations in Parkin and PINK1 on mitophagy are being examined in more physiologically related setting with the aid of induced pluripotent stem cell (iPSC)-derived neurons from PD patients. Parkin translocation to mitochondria was arrested in DA neurons derived from PD patients carrying PINK1 mutation^50^. The expression of several mitophagy-related genes was altered in DA neurons derived from Parkin knockout isogenic iPSCs^51^. Aberrant accumulation of phospho-ubiquitin and aggregated mitochondria have been demonstrated in neurons differentiated from iPSCs carrying Parkin or PINK1 mutations. Of note, the majority of phospho-ubiquitin was detected in TH-positive neurons, but not in TH-negative neurons, highlighting the cell type-specific mitophagy defects^52^. In idiopathic PD, impaired mitochondrial clearance was also observed, but not in all the iPSC clone-derived neurons, which could be attributed to the heterogeneity of PD pathogenesis^53^. In addition to phospho-ubiquitin, abnormalities of other mitophagy-related proteins have been recorded utilizing patient iPSC-derived neurons. It was found that Miro, a substrate of Parkin, was resistant to proteasomal degradation induced by mitochondrial depolarization, interferencing mitochondrial mobility and initiation of mitophagy in PD patients with LRRK2 G2019S mutation and sporadic PD patients^7^. Mutation in α-synuclein also causes Miro protein accumulation, inhibiting mitophagy in PD patient neurons^16^. Consistently, co-localization of mitochondria with lysosomes and clearance of mitochondria were significantly reduced in iPSC-derived neurons carrying Miro mutation^54^. mt-Keima is a mitochondria-targeted fluorescent protein, which is sensitive to pH changes but resistant to degradation mediated by lysosomal proteases. When mitophagy occurs, mt-Keima is engulfed by acidic lysosome where the excitation of mt-Keima shifts to a longer wavelength compared with mitochondrial physiological environment. The ratio change in excitation wavelengths of mt-Keima has been utilized to quantify mitophagy^54,55^. With the aid of mt-Keima, mitophagy defects were confirmed in iPSC-derived neurons with Parkin/PINK1 mutations by independent groups^56,57^. However, the fidelity of the iPSC-derived neuron model system to reveal the mitophagy impairment should be interpreted cautiously. Inhibition of PINK1-dependent mitophagy dramatically compromises iPSC reprogramming^58^. In addition, mitophagy mediates the metabolic switch between glycolysis and mitochondrial oxidative phosphorylation, which is critical during cell differentiation^59^. It is therefore possible that compensatory mechanism may have been developed during the conversion process of patient somatic cells to neurons. Alternatively, cells with substantial mitophagy defects may have failed to be transformed into neurons. Thus, the mitophagy dysfunction revealed in the patient iPSC-derived neurons may not faithfully recapitulate neuronal mitophagy status in the patient brain. Despite the limitations, studies in iPSC-derived neurons provides unequivocal evidence of mitophagy defects in PD.

#### Parkin-independent mitophagy

Accumulating evidence suggests that Parkin is not canonically essential for all mitophagy processes. Receptor-mediated mitophagy centers around the ability of LC3-interacting region (LIR) motif that are identified in certain protein receptors to allow binding to LC3 for mitophagy induction, regardless of the presence of Parkin^60–62^. AMBRA1 utilizes LIR motif to facilitate interaction with LC3, and mitophagy induction under conditions lacking Parkin^61^. FUNDC1, initiates mitophagy independent of Parkin, whereby evidence points towards its LIR domain association with LC3 as well as MARCH5 and ULK1 interactions for inducing hypoxia-related mitophagy^62,63^. Bcl-2/adenovirus E1B 19kD-interacting protein 3 -like proteins (BNIP3L), also known as NIX is an outer mitochondrial membrane protein equipped with an LIR near its N-terminus to perform Parkin-independent mitophagy^64,65^. A study using PD patient cells identified that despite carrying non-functional Parkin, BNIP3L/NIX-mediated mitophagy had maintained mitochondrial function, suggesting the PD-relevant neuroprotection^66^. A slew of other emerging proteins acting as receptor-mediated mitophagy interactors have been added on to this growing list, including STX17^67^, FKBP8^68^, Bcl2-L-13^39^ and PHB2^69^. Cardiolipin is a lipid that triggers mitophagy through similar mechanisms mentioned in the previous receptors, externalizing from IMM to OMM under mitochondrial stress and binding to LC3, which upon reaching a critical threshold, trigger the induction mitophagy in a neuronal setting^70^. The sphingolipid ceramide, capable of anchoring autophagolysosomes to mitochondrial membranes via LC3 interactions^71^ has also been suggested in parkin-independent, lipid-mediated mitophagy. Ubiquitin-mediated mitophagy was identified through PINK1’s ability to trigger mitophagy through NDP52 and optineurin regardless of Parkin’s presence, rewriting Parkin’s role from an essential regulator to a signal amplifier^23^. Other E3 ligases aside from Parkin, such as MUL1 was found to interact with mitofusin (Mfn) during mitophagy induction at damaged mitochondria, and in Parkin-independent pathway leading to mitochondrial ubiquitination and mitophagy^72,73^. Another E3 ligase, SIAH1 is recruited to mitochondria by Synphilin-1 to promote PINK1-dependent mitophagy via mitochondrial ubiquitination in a Parkin-independent manner^74^. Collectively, these observations suggest a complex mitophagy pathway involves an intricate compensatory network that responds to various homeostatic and pathological conditions (Fig. 1). Although there is little evidence of Parkin-independent mitophagy being involved in PD, these alternative mitophagy mechanism, such as MUL1- or NIX-involved mitophagy as discussed above, may be utilized to correct the mitophagy defects to rescue neurodegeneration in PD.

### Reactive oxygen species

Reactive oxygen species (ROS) are highly reactive derivatives of molecular oxygen generated as by-products of cellular respiration. Common ROS include superoxide anion radical (O_2_^●−^), hydroxyl radicals (OH^●−^), and hydrogen peroxide (H_2_O_2_). ROS are normally sustained at low levels under redox homeostasis conditions^75^. Maintaining ROS within balance is supported by antioxidant systems, with endogeneous enzymatic antioxidants, such as superoxide dismutase (SOD)^76^, catalase (CAT)^77,78^, glutathione peroxidase (GPx)^79^ and thioredoxin (Trx)^80^, as well as nonenzymatic antioxidants including vitamins such as vitamin C^81^, vitamin E^82^ which collectively counter ROS overproduction within cells directly or indirectly^83^. ROS are predominantly the result of excessive electron slippage in the electron transport chain (ETC) exceeding the various antioxidant systems, and capable of readily oxidizing other molecules (proteins, DNA and lipids), causing a wide-range of effects^84–87^. ROS oxidizing effects on proteins include protein carbonylation, the most well-documented form of protein oxidation, whereby protein side chains are targeted, resulting in loss of protein functions^84^. ROS are the primary endogenous damaging agent of DNA, with these oxidative reactions incurred through oxidation of DNA bases^85^. ROS compromise the structural and dynamic properties of lipid membranes by altering their fluidity and permeability^86^. Lipid peroxidation is the common term used to describe the chain reaction induced by ROS targeting lipids leading to key breakdown molecules malondialdehyde (MDA) and 4-hydroxy-2-nonenal (HNE)^87^. These intermediary compounds may react with DNA and proteins to form adducts in a neurodegenerative setting^88,89^. Lipid peroxidation by-product HNE triggers AMPK/mTORC pathway^90^ and JNK signal transduction pathway^91^ subsequently leading to autophagy activation^92,93^. Cardiolipin, a phospholipid and important mitophagy mediator in the inner membrane of the mitochondrial lipid bilayer, was identified to be specifically susceptible to lipid peroxidation^94,95^. Oxidation of Cardiolipin leads to the generation of reactive lipid mediators, including HNE and epoxide-containing cardiolipin^94,95^. The mitochondrial network as a whole is a critical target of 4-HNE, with neuronal autophagy flux identified to be modulated by 4-HNE in a concentration-dependent manner likely through direct modification of autophagy proteins^96^. Considering the effects of lipid peroxidation on autophagy, cardiolipin and mitochondrial protein qualities, it stands to reason the possibility that ROS-induced lipid peroxidation could eventually regulate mitophagy. Despite the classic dogma of ROS and reactive nitrogen species (RNS) being widely considered as harmful to cellular structures, their role is dynamic, serving pleiotropic intracellular signal transducers of inter-connected processes, including development processes^97,98^, metabolic networks^99^ and autophagy^100^.

#### Evidence of ROS in PD

ROS are signalling molecules that maintain several physiologic functions. When the physiologic compensation fails to keep ROS at appropriate levels, the resulting oxidative stress will damage cellular macromolecules and promote cell death^101^, leading to neurodegeneration. Various in vitro and in vivo studies in PD models have shown ROS-induction are critical in the pathophysiologic mechanisms of PD genetic mutations (PINK1 and Parkin^102^, DJ-1^103^ LRRK2^104^ etc) and mitochondrial dysfunction^105^. Furthermore, dopamine auto-oxidation promotes neurotoxicity in DA neurons^10^. In post-mortem studies, there is evidence of reduced mitochondrial electron transport chain activity, and an increase in iron in SN compared to age-matched controls^106,107^. Iron is known to raise ROS production through generating hydroxyl radicals, leading to α-synuclein-mediated reactive species formation in SN via Fenton reaction and redox-active iron accumulation in neuromelanin granules in SN^25^. DA neurons in the SN are more susceptible to excess ROS generation as a result of their distinct cellular structure (relatively increased size and complexity) and biochemical activities (unique Ca^2+^ pacemaking activity)^108,109^.

#### Evidence of ROS in mitophagy

There is considerable evidence suggesting a key role of ROS in mitophagy. Light-induced activation of mitochondria-targeted photosensitizer may cause selective ROS-mediated damage to a subset of mitochondria and subsequently trigger mitophagy in both cell lines^110^ and rodent neurons^111^. In vitro studies have demonstrated that mild and transient oxidative stress can trigger mitophagy but not non-selective autophagy^40^. Downregulation of mitochondrial fusion can spatially isolate damaged mitochondria for efficient removal by mitophagy, highlighting the selectivity of oxidative conditions in a ROS signalling cascade that dominantly triggers selective mitophagy^40^. Our own studies found that inhibition of ROS burst attenuated mitophagy at a couple of stages, i.e. PINK1-dependent Parkin translocation to mitochondria^102^ and elimination of mitochondria through autophagy^112^. Interestingly, chronic low-dose treatment of mitochondrial uncoupler failed to stimulate ROS upsurge and Parkin translocation to mitochondria despite bringing PINK1 protein to a comparable level as acute high-dose treatment. Given the indispensable role of PINK1 in the initiation of mitophagy, there is the possible modification of ROS on PINK1 in the process of mitophagy^102^.

## The bridges between ROS and Mitophagy

Being the primary source of cellular ROS and the central focus of mitophagy, mitochondria are the crux of a number of signalling pathways linking ROS and mitophagy^112,113^. ROS are a double-edged sword, possessing bidirectional impacts on autophagic flux. Generally, excessively high levels of ROS specifically trigger general autophagy over mitophagy^40^. In contrast, moderate levels of ROS may trigger mitophagy likely through the activation of specific signalling pathways and redox signalling^40,114^ with mitophagy in turn possessing neuroprotective effect on disease progression^66,115^. Collectively, whilst ROS as a signalling molecule and protective mitophagy are physiologically critical, prolonged dysregulation of mitophagy flux^7,16^ and/or excessive ROS levels^9,108^ are often detrimental for neuronal status, including within PD context. Identifying the bridges linking ROS and mitophagy is crucial for establishing their roles in PD pathogenesis and postulate potential therapeutic avenues using these interplays.

### Signaling pathways

#### NF-κB

Nuclear factor kappaB (NF-κB) represents a family of five transcription factors, including NF-κB1 (p50/p105), NF-κB2 (p100/p52), RelA (p65), RelB, and c-Rel^116^. Critical in inflammation, NF-κB family members upon activation through canonical and/or noncanonical pathways, may rapidly translocate to the nucleus and mediate transcription of target genes by binding to κB enhancer^117^. Substantial evidence suggests that ROS (H_2_O_2_) acting upstream of this master regulator of redox balance, NF-κB, releases its NF-κB inhibitor (IκB) via H_2_O_2_ oxidation leading to NF-κB activation^118^. Recently, it was found that NF-κB may promote mitophagy through inducing p62 expression to attenuate mitochondrial damage triggered by NLRP3-inflammasome activator. Macrophage death was aggravated by inhibition of mitophagy through ablation of p62, or pathologically compromised ‘NF-kB-p62-mitophagy’ pathway. The same study suggested that NF-κB, through this anti-inflammatory pathway, directly affects Parkin-mediated mitophagy^119^. A study by Duan et al. identified several NF-κB-binding sites within the PINK1 promoter and demonstrated that NF-κB overexpression or administration of NF-κB activator upregulated PINK1 at transcription level^120^. TRAF6, an E3 ligase that acts as a transducer of the NF-κB pathway, and its related NF-κB activation may activate PINK1 cytosolic form to promote non-selective mitophagy through stabilization by enhanced Lys-63-linked ubiquitination^121^. In addition, NF-κB may promote RIPK1 translocation to the mitochondria where it forms a complex with PINK1 and phosphoglycerate mutase family member 5 (PGAM5) that stabilizes and activates PINK1, eventually inducing mitophagy^122^.

#### p38 MAPK

p38 MAPK belongs to the family of mitogen-activated protein kinases (MAPKs) and is responding to stress stimuli, including inflammatory cytokines and oxidative stress^123^. Under the canonical pathway of activation, ROS signalling by oxidative stress specifically oxidize antioxidant protein thioredoxin (TRX) and disassociate it away from the critical component ASK-1, allowing for ASK-1 dimerization and autophosphorylation, activating p38 pathway^123^. MAPK14, one of the four p38 isoforms, and its upstream signalling pathways are identified to be required in mammalian cells for both starvation- and hypoxia-induced mitophagy, but not macroautophagy, demonstrating the selectivity of this pathway^124^. A recent study by Qu et al. identified that inhibition of MAPK/p38 compromised the redistribution of Parkin in Parkin/PINK1-dependent mitophagy, with ROS identified to be a critical mediator upstream of this mitophagy pathway^112^. Additionally, our own study identified that inhibition of p38 signalling pathway halted mitophagy progression driven by ROS even after Parkin relocated to mitochondria, further corroborating the hypothesis of MAPK having a significant influence on upregulating ROS-mediated mitophagy^113^.

#### mTOR

Mammalian target of rapamycin (mTOR) is a serine/threonine protein kinase, and it inhibits autophagy through a complex interplay and mTOR dysfunction can lead to apoptosis of DA neurons in PD animal models^125^. ROS may regulate autophagy/mitophagy through the PI3K/AKT/mTOR signalling pathway^126,127^. The role of MTORC1 in autophagic removal of mitochondria is confirmed with TSC/MTORC1 identified to be essential regulators for downstream mitophagy induction^128^. Additionally, there is a possible influence on FoxO regulated Parkin/PINK1 axis, with inhibition of mTORC1 identified to precede FoxO-dependent mitophagy upregulation^129^.

#### Nrf2-related pathways

Nrf2 (nuclear factor erythroid 2-related factor 2) is a redox-regulated transcription factor, a core element in the Nrf2-Antioxidant Response Element (ARE) related pathways. Their regulation can be either Kelch-like ECH-associated protein 1 (Keap1)-dependent or Keap1-independent (via phosphorylation by Casein kinase II, protein kinase C, glycogen synthase kinase 3β etc)^130^. These pathways are identified to mediate oxidative stress response and are established to be dysregulated in aging and neurodegenerative diseases, including PD^130^. Nrf2 pathway activation in PD was observed at a systemic level, likely to counteract oxidative stress^131^. Oxidative stress may deprive Keap of its ability to ubiquitinate Nrf2 by modifying key cysteinyl residues leading to nucleus transport of Nrf2. Accumulated Nrf2 subsequently activates a body of antioxidant enzymes, acting as a critical master regulator for a broad set of oxidative stress responses^118^. Oxidative stress triggered Nrf2 binds to ARE located in the p62 promoter, with p62 possibly activating Nrf2 and driving its own transcription in a positive feedback cycle^132^. p62-mediated mitophagy inducer (PMI) disrupts the Nrf2-Keap1 interaction and induces mitophagy independently of Parkin/PINK1^133^. Alternatively, another study suggests that p62, in an Nrf2-dependent manner, recruits two subunits of a cullin-RING ubiquitin (Keap1 and Rbx1) to mitochondria, promoting mitochondrial ubiquitination and subsequently mitophagy in a parkin-independent manner^134^.

#### SIRT

**Fig. 2:**
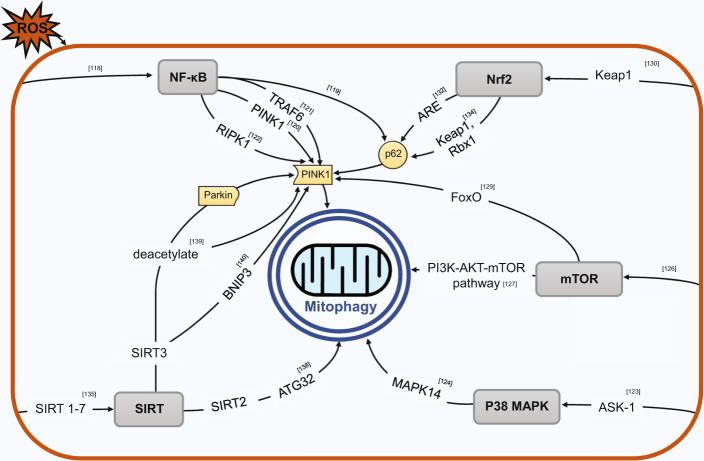
Core signalling pathways bridging ROS and Mitophagy. ROS function upstream of intracellular signalling pathways which involve several key mediators (NF-κB, p38 MAPK, mTOR, Nrf2, SIRT) to regulate mitophagy.

Sirtuins, are a family of nicotinamide adenine dinucleotide (NAD)-dependent histone deacetylases, which respond to ROS-activated NAD^+^ function to regulate numerous antioxidant and redox signalling pathways through transcription factors (including Nrf2, p53, NF-κB, FOXO, PGC-1a) and several molecules of antioxidant response element (ARE)^135^. Sirtuins relevancy in PD context includes a variety of SIRTs appearing to alter mitophagy in PD models in addition to their expression dysregulated in PD patient samples. For instance, decreased SIRT3 was found in the fibroblasts from PD patients and SIRT5 was accumulated in idiopathic PD fibroblast cells, with both these sirtuins being mitochondrial proteins^136^. The relationship between sirtuins (SIRT1-7) and ROS, is uniquely complex and contested, with current evidence indicating that SIRT4 conditionally upregulates or suppresses ROS; SIRT1, SIRT3 and SIRT5 function against ROS; SIRT2, SIRT6, and SIRT7 mediates critical oxidative stress genes and mechanisms^135^. SIRT1 influences Parkin translocation to mitochondria and is closely tied to alterations NAD + /NADH ratio in studies observing SIRT1 influence on mitophagy^137^. In addition, SIRT2-mediated mitophagy regulated via ATG32 is identified to be essential in α-synuclein toxicity in yeast samples^138^, implicating this ROS-mitophagy interplay in PD context. SIRT3, known for downregulating ROS through modulation of several critical enzymes, was identified to be key for inducing Parkin/PINK1-mediated mitophagy, likely through SIRT3 ability to interact and deacetylate both PINK1 and Parkin directly or indirectly via FOXO3a^139^. Interestingly, another study identified SIRT3 effect of inducing mitophagy by upregulating BNIP3 expression, this mitophagy modulating activity further tied with ERK-CREB signalling pathway^140^ (Fig. 2).

### PD-related mutations/genes

#### Parkin/PINK1

Mutations in the *parkin* and *PINK1* account for the most common causes of autosomal recessive early-onset Parkinson disease (EOPD). Early studies exploring functions of Parkin and PINK1 revealed that defects in Parkin or PINK1 result in enhanced ROS production in mouse brain^141^ or patient fibroblast^142^, suggesting that Parkin and PINK1 may confer protection to neurons by attenuating ROS-related neurotoxicity. The mechanism of this protection at least partially lies in the role of Parkin/PINK1-mediated mitophagy that is progressively unravelled and refined. Overexpressed *parkin* in cultured cell lines can eliminate the entire mitochondrial network in cells, eradicating detrimental factors released from damaged mitochondria^143^. Although dramatic mitophagy is not practicable in neurons, evidence of ROS inhibition through mitophagy in a physiological context has been garnered. Parkin/PINK1-dependent mitophagy was shown to attenuate NLRP3 inflammasome activation and mitochondrial ROS production, eventually reducing apoptosis in epithelial cells and renal injury in mice^144^. AMPKα2 may interact with phosphorylated PINK1 and trigger Parkin recruitment and subsequent mitophagy, leading to reduced ROS production and apoptosis of cardiomyocytes^145^. Inhibition of PINK1 accumulation on mitochondria by morphine led to mitophagy defect and excessive ROS accumulation in spinal cord neurons^146^.

#### α-synuclein

Mutations in the gene encoding *α-synuclein* (*SNCA*) cause autosomal-dominant PD through point mutations, including A53T, E46K, H50Q etc, as well as copy number variations (duplication or triplication)^147^. Neurodegenerative effects of α-synuclein in PD have been increasingly tied to α-synuclein reciprocal relationship with mitochondrial dysfunction^148^, with a possible link to PINK1-related autophagy/mitophagy^16,149^. α-synuclein role in ROS includes its accumulation on mitochondria via TOM20 leading to an excessive generation of ROS, with pathogenic a-synuclein-TOM20 interaction confirmed in PD post-mortem samples^150^. α-synuclein delays mitophagy by targeting the N-terminus of Miro, leading to excessive and abnormal Miro accumulation on the mitochondrial surface^16^. Upregulated production of ROS and reactive nitrogen species (RNS) are present in iPSCs carrying *α-synuclein* A53T mutation relative to isogenic control lines^151^. *α-synuclein* E46K mutation was also identified to result in oxidative stress accumulation in SN DA neurons, likely increasing neuronal vulnerability towards mitochondrial impairment by mitochondrial toxins^152^. Both A53T and E46K mutations promoted mitophagy through increasing α-synuclein accumulation on mitochondria, primarily through cardiolipin externalization to the OMM^153^.

#### DJ-1

*DJ-1* gene has been identified to be mutated in autosomal recessive PD. DJ-1 protein acts as a neuroprotective factor by directly eliminating hydrogen peroxide, while cells expressing DJ-1 carrying PD-related mutations are sensitive to oxidative effects^10,154^. Interestingly, as a sensor for oxidative stress, DJ-1 protein is susceptible to the formation of adducts with PD-relevant dopamine^155^. This relationship is theorized as a key susceptibility factor within PD-relevant A9 neurons in SN region that contain dopamine susceptible to ROS-induced adduct formation by DJ-1, further supported by in vivo evidence of elevated α-synuclein aggregates in *DJ-1* deficient mice leading to the increased oxidized form of DA^10,156^. Additionally, a previous study identified that *DJ-1* overexpression rescued DA neurons against neurotoxicity, including oxidative stress, by enhancing extracellular signal-regulated protein kinase 1/2 (ERK1/2)-dependent mitophagy^115^. This is in addition to the likely reciprocal interplay with Parkin/PINK1, as DJ-1 deficiency was identified to, in a ROS-dependent manner, promote Parkin recruitment and mitophagy, while mitochondrial DJ-1 level being regulated by Parkin/PINK1^103^. Hence, while the exact mechanism is yet to be fully elucidated, DJ-1 is believed to influence Parkin/PINK1 mitophagy in a ROS-dependent manner.

#### LRRK2

LRRK2, is a multifunctional enzymatic (kinase, GTPase etc), scaffolding protein^157^. *LRRK2* G2019S is a common genetic cause for familial and sporadic PD in caucasian populations^157^, whereas in Asian population, *LRRK2* S1647T, R1628P, and G2385R variants are associated with increased risk and lowered onset age of PD^158^. iPSC-derived neurons carrying G2019S mutation was shown to upregulate the expression of key genes related to oxidative stress-response and α-synuclein levels^159^. Our group have shown that quenching ROS through either genetic manipulation (expressing peroxiredoxin-3)^160^ or administration of chemical antioxidant^161^ effectively rescued PD phenotypes in neuronal and Drosophila models, suggesting that ROS play a crucial role in LRRK2 pathogenesis. *LRRK2* mutations possess a complicated relationship with mitophagy, as the same G2019S mutations have demonstrated various mechanisms of mitophagy alterations, from increasing mitophagy through histone deacetylase activation^136^, or in contradiction, to decreasing mitophagy^162^ probably through compromised Miro removal^7^, sirtuin activity^163^ or aberrant RAB10 phosphorylation^164^. Regardless of the uncertainty of specific regulation, which is likely context-dependent, it can be agreed that *LRRK2* mutations, particularly G2019S, result in aberrant mitophagy within the PD context, likely contributing to its increased mitochondrial vulnerability and overall neurodegeneration^156^.

#### GBA

Glucocerebrosidase (*GBA1*) gene mutation is associated with a 20- to 30-fold increased PD development risk^165^. L444P mutation in *GBA* has been established to induce mitochondrial dysfunctions, including altered mitophagy activation, autophagy flux and ROS levels^166^. Specifically, L444P mutation in *GBA* has been identified to inhibit two critical areas related to mitophagy, namely mitochondrial priming and autophagic removal of the organelle^167^. Mitophagy induction is identified to enhance transcription factor EB (TFEB) expression leading to increased GBA1 expression. The involvement of mitophagy in lysosomal biogenesis suggests a positive feedback loop, with GBA mutations inducing dysfunctions within this system^167^. In addition, dysfunctional mitophagy and excessive oxidative stress have been identified in post-mortem tissue of PD patients with *GBA* mutations^167^, further linking the effects of GBA on ROS-mitophagy interplay in PD context.

In our review, the selection of genes we focus on reflect the most well-documented PD-related genes with regards to how they are functionally linked to mitophagy and ROS. However, genome-wide association study (GWAS)^168–170^ have revealed a host of relatively novel risk loci and their genes, with limited but emerging evidence that some could be implicated in both mitophagy and ROS, including VPS13C^171^ and SREBF1^172^. We speculate that evidence of genetic variants in PD relevant context could increase in the coming years.

We have highlighted a few PD genes that are associated with both aberrant ROS accumulation and defective mitophagy. The body of the evidence above clearly proves that the abnormalities in these two aspects are universal and critical in PD. However, more studies are warranted to determine which factor is the major culprit for PD pathogenesis and how the entanglement of these two factors contribute to PD pathogenesis. Therefore, both factors have to be taken into account to deliver effective therapy when designing a strategy to tackle PD.

In addition, although the overwhelming evidence supports the agonistic role of ROS in mitophagy through activating various signalling pathways, there are reports showing antioxidative molecule(s) and/or compound(s) may promote mitophagy. For instance, KH176, a chemical derivative of a water-soluble form of vitamin E, enhanced mitophagy in neurons from subjects with or without Parkin mutation, suggesting its pro-mitophagy effect in a Parkin-independent manner^173^. KH176 exerts its anti-oxidative effects through direct interaction with peroxiredoxins^174^, while peroxiredoxin 3^175^ and peroxiredoxin 6^176^ have been found to be involved in mitophagy. Another study showed that mitochondria-targeted antioxidant, MitoQ rescued mitophagy, mitochondrial dysfunction and apoptosis through Nrf2 and PINK1 rather than its direct antioxidative effect^177^. Thus, the paradoxical pro-mitophagy effect of antioxidant may be attributed to its specific effects on mitophagy-related signalling pathways or molecules instead of its role in suppressing ROS. In addition, ROS may directly oxidize ATG3 and ATG7 to prevent LC3 lipidation, eventually impairing autophagy^178^. Given that Atg3^179^ and Atg7^180^ also play indispensable roles in mitophagy, it is therefore conceivable that antioxidants may suppress the oxidation of these autophagy-related proteins to promote mitophagy in a context-dependent manner. The success of antioxidant in promoting mitophagy indicates the potential of the therapeutic strategy for PD by promoting mitophagy while inhibiting ROS.

## Therapeutic avenues utilizing the ROS-mitophagy link

Clinical trials throughout the decades of antioxidants (creatine^181^, vitamin E^182^, coenzyme Q_10_^183^, etc) in PD have not provided conclusive evidence that they are neuroprotective. One possible reason we postulate for the failure of antioxidants could be that blockage of signalling pathway necessary for mitophagy activation by undue inhibition of ROS is unlikely to be optimal against neurodegeneration. Promoting mitophagy by direct induction of ROS may not be ideal strategy either given the high volume of evidence showing the detrimental effects of ROS on cellular health, on account of its ability to exacerbate oxidative stress damage in PD^9^.

### Existing compounds/drugs with effects on ROS and mitophagy

Targets in the downstream signalling of ROS which can simultaneously activate protective mitophagy, while minimizing the harm of ROS may be potentially utilized in PD therapeutics. Here, we highlight some promising chemical compounds/drugs that target the various signalling pathways that may be involved in mitophagy. These chemical compounds/drugs have been approved or under investigation for other diseases, so that the safety concern may be circumvented.

Melatonin (N-acetyl-5-methoxytryptamine) has been shown to increase phosphorylation of Akt and NF-κB, leading to PINK1-dependent protective mitophagy^184^. Melatonin may also upregulate NRF2-induced mitophagy to protect against neuronal apoptosis in subarachnoid haemorrhage (SAH)^185^. The ROS levels, initially increased by high glucose conditions and necessary in order to stimulate mitophagy via Akt and NF -κB pathways, were subsequently attenuated by melatonin-induced PINK1-dependent mitophagy^184^. Melatonin has been well-documented to function against ROS and RNS, but may have pro-oxidant capabilities under specific conditions^186^. Existing evidence points toward melatonin’s role as an antioxidant showing systemic therapeutic benefits in PD-relevant clinical trials (dyskinesia, sleep disorder)^187^, which combined with amphiphilic nature allowing for BBB diffusion further encourages therapeutic focus revisiting melatonin as a mitophagy agonist with NF-κB and Nrf2 activating effect mentioned prior^187^.

Pioglitazone, an anti-diabetic drug for Type 2 diabetes mellitus (T2DM) under the category of thiazolidinediones (TZDs), increases PINK1 expression via NF-κB activation and enhances mitophagy. It protects against mitochondrial dysfunction induced by toxins and reduces ROS in a chronic kidney disease study^188,189^. Pioglitazone also improved phenotypes (impaired locomotion, DA neurodegeneration) in rat PD model^190^. Clinical cohort studies showed a 30% reduced risk of PD among diabetes treated with TZDs^191,192^.

Rapamycin, an FDA-approved compound, induces autophagy by binding and inhibiting mTORC1^193^, and has been tested as a disease-modifying agent in experimental models of PD and neurodegenerative diseases^194^. It also promotes Parkin/PINK1 mitophagy, likely via p62, to restore mitochondrial homeostasis^195,196^. Rapamycin’s effect on ROS appears to be conditional. It may downregulate intracellular ROS via Nrf2 pathway, glutathione and SOD^197,198^. Nevertheless, it is also able to increase ROS levels, probably through c-Jun and endoplasmic reticulum (ER) stress pathway in certain circumstances^199^. Metformin, an mTOR inhibitor, activates mitophagy via signalling pathways including AMPK-Nrf2 as well as SIRT3 pathway^200,201^. It has been shown to upregulate mitophagy in a recent randomized controlled clinical trial in type 2 diabetics^202^. Metformin has been found to downregulate ROS production^203,204^. A longitudinal cohort study in an aging population with diabetes demonstrated a lower incidence of neurodegenerative diseases in those on long-term metformin therapy^205^. Spermidine, a polyamine with anti-aging properties, induces mitophagy through several signalling pathways, including modulation of the mTOR axis via ATM-dependent Parkin/PINK1 pathways^206,207^. Of note, PD gene PARK9 encoded protein ATP13A2 is a lysosomal exporter of polyamine (putrescine, spermidine, and spermine)^208^. Spermine is pumped by ATP13A2 into the cytosol and subsequently absorbed by mitochondria. The polyamine transport activity of ATP13A2 is responsible to counter the mitochondrial oxidative stress^209^. Mitochondrial defects observed in *C. elegans* carrying a mutation in CATP-6 (a *C. elegans* ortholog of ATP13A2) was rescued via mitophagy induction^210^. In another study, spermidine was able to rescue behavioural deficits in the PD *C. elegans* model via the Parkin/PINK1 equivalent pathways^207^. Taken together, these studies suggest that spermidine may have neuroprotective effect on PD with ATP132A being a key component. Despite spermidine-generated ROS being a possible upstream signal in activating the latter ATM pathway^206^, spermidine appears to act as direct ROS scavengers, resulting in inhibition of mitochondrial ROS production^211,212^. Salidroside, a plant extract, has been shown to protect DA neurons in PD models through enhancing mitophagy with its bioactive effects on DJ-1/Nrf2 pathway being the likely mechanism^213^. Consistently, studies revealed neuroprotective effects of salidrosides in pre-clinical trials in Alzheimers disease, stroke and PD. A number of these studies emphasized its safety and substantial bioactive effects on the regulation of oxidative stress^214^.

Tomatidine, a natural compound with antiaging properties activates Nrf2-SKN-1 pathway, resulting in the *C. elegans* homologue induction of PINK-1/BNIP3/Nix-mitophagy that is suspected to preserve cell function by clearing damaged or dysfunctional mitochondria^215^. Tomatidine may activate mitophagy via Nrf2-SKN-1 pathway or TRAF6. Meanwhile, it may have a negligible or mild stimulating effect on ROS production^215,216^. Although evidence is limited, Ursolic and oleanolic acids, natural triterpenoid compounds widely found in plants, have been shown to induce Parkin-independent mitophagy, possibly concurrent with activation of AKT/mTOR and Nrf2/ARE pathways, with the ROS induction suspected to be an upstream factor for mitophagy induction for both triterpenes^217^.

Honokiol, an agonist of SIRT3 known for anti-inflammatory and antitumor effects, promotes mitophagy and mitochondrial dynamics in vitro in an SIRT3-dependent manner via the AMPK-PGC-1α signalling pathway, and this neuroprotection has been validated in vivo^218^. Honokiol’s effect of downregulating ROS in this study^218^ is consistent with existing in vitro and in vivo evidence highlighting its inhibitory effect on ROS production, likely in an SIRT3-dependent manner^219,220^.

Liraglutide, an anti-diabetic drug, is a glucagon‑like peptide‑1 receptor agonist that elevates the levels of SIRT1 in a dose-dependent manner and is able to trigger autophagy. It can augment Parkin expression, leading to Parkin-mediated mitophagy activation^221^. The SIRT1/Parkin/mitophagy pathway activation is protective, downregulating cellular oxidative stress^221^. Similar observations of ROS inhibition by liraglutide has been consistently observed^222,223^. Liraglutide shows good BBB permeability and neuroprotective effects in PD animal models^224^. We await the results of its ongoing trial in idiopathic PD, which should be completed soon. (ClinicalTrials.gov Identifier: NCT02953665).

We discuss here the compounds/drugs that regulate mitophagy with emphasis on ROS-related signalling pathway(s). As for the modulators of mitophagy reviewed elsewhere, most of the targets are localized on mitochondria^225,226^. Therefore, modulation of these targets may have profound impacts on mitochondrial functions, including ROS homeostasis. For instance, Ubiquitin-specific protease 30 (USP30) removes ubiquin attached by Parkin to the OMM substrates, attenuating subsequent mitophagy. On the other hand, reduction in USP30 level rescues mitophagy caused by Parkin/PINK1 mutation. Based on its influence on mitophagy, USP30 inhibitors have been developed to promote mitophagy as a potential therapeutic option for PD^227,228^. However, USP30 is also involved in the regulation of the import of intramitochondrial proteins, including subunits of electron transport chain proteins such as Complex-I^229^. Manipulation of USP30 may cause aberrant ROS generation by interfering the proper functions of the respiratory chain. Thus, the effects of USP30 or other mitophagy regulators on ROS production have to be assessed and the benefit of neuroprotection through mitophagy and the potential detrimental effect of ROS dysregulation has to be weighed.

Maximizing the potential therapeutic benefits of these compounds in Table 1 requires factoring in their effects on ROS levels. Among them, a few compounds may potentially upregulate ROS, having a negative impact on cell survival. When applied in PD, these drugs should be coupled with ROS inhibitors (general antioxidants or mitochondrial-targeted antioxidants) necessary to negate any harmful oxidative effects. Most compounds listed have apparently null effects or downregulate ROS levels, whereby their use in combination with ROS inhibitors may be considered to synergistically improve therapeutic performance^230^. We reason that mitophagy activation in PD relevant therapeutics should simultaneously avoid provoking oxidative stress, while maintaining their neuroprotective effects of downstream signalling on mitochondrial homeostasis, particularly mitochondrial turnover through autophagy-lysosomal pathway.

**Table 1:** Relevant compounds for consideration of ROS-mitophagy link.

Compound	Target mitophagy pathway	Effect on ROS levels	References
Melatonin	Upregulation of NF-κB (preceded by Akt) and Nrf2 to induce mitophagy	ROS reduced	^184–187^
Pioglitazone	NF-κB to induce mitophagy	ROS reduced	^188–192^
Rapamycin	mTOR inhibition to induce Parkin-mediated mitophagy	ROS reduced in majority of studies, raised in few studies under specific conditions	^193–199^
Metformin	mTOR inhibition and SIRT3 upregulation to induce Parkin-mediated mitophagy	ROS reduced	^200–205^
Spermidine	mTOR axis modulation to induce Parkin mitophagy	ROS reduced overall, early ROS-generation acting as an upstream signalling factor	^206–212^
Tomatidine	Activated Nrf2-SKN-1 pathway to induce PINK-1/Bnip3/Nix-mitophagy	Mild or no effect	^215,216^
Ursolic andOleanolic acids	Activate AKT/mTOR and Nrf2/ARE pathways to induce Parkin-independent mitophagy	Early ROS-generation acting as an upstream signalling factor	^217^
Honokiol	Trigger SIRT3 (via the AMPK-PGC-1α) pathway to induce mitophagy	ROS reduced	^218–220^
Liraglutide	Increase SIRT1 levels leading to Parkin-mediated mitophagy activation	ROS reduced	^221–224^

Some of these compounds are currently undergoing pre-clinical and clinical trials (i.e. Liraglutide, Salidroside, and Melatonin) in PD, further encouraging the efforts in drug repurposing. Therapeutic usage of these compounds could implemented in a targeted manner or in combined multidrug regime with existing pharmacological (carbidopa-levodopa, pramipexole etc) and non-pharmacological approaches (physical, occupational, speech therapies etc)^6^.

Drug repurposing, utilizing various degrees of pre-existing clinical efforts covering efficacy and safety efforts, offsets the high cost and lengthy timeline of de novo drug discovery^231^. However, drug repurposing comes with issues that should be taken into consideration, including but not limited to intellectual property considerations and high chance to fail in clinical trials^231,232^. The ROS inhibitors as mentioned earlier (creatine^181^, vitamin E^182^) have yet to be proved as effective repurposing drugs in PD treatment.

### Potential target-based screening for mitophagy activator

**Fig. 3:**
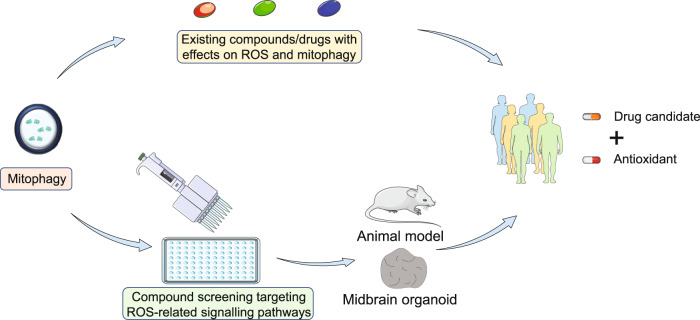
PD therapeutic strategies targeting ROS-mitophagy link. Existing compounds/ drugs could be utilized to induce mitophagy while their direct/indirect effects on ROS are evaluated. Alternatively, compound screening of mitophagy activator targeting ROS-related signalling pathways, may yield hits that potentially reverse the pathogenesis of PD. The hits of the screening should be validated in multiple PD model systems given the limitations of the current disease models. The drug candidates identified from either strategy will be recommended to enter potential clinical trial with antioxidant therapy to aid removal of potential ROS-related detrimental effects.

Alternatively, potential therapeutic compounds for PD may be discovered *de novo* from the perspective of mitophagy. A recent study was conducted using an imaging-based drug screening in patient iPSC-derived neurons carrying Parkin or PINK1 mutation. Among the 320 compounds screened, 73 hits were found to promote mitophagy with two candidates attenuating ROS and cell death^53^. Large-scale screenings are needed to identify more potential compounds which can be further validated and taken to clinical trials. It should be noted that neurons are usually cultured in B27 supplemented neurobasal medium, which contains antioxidant to promote neuronal survival^233^. Potential oxidative stress caused by compounds may not be revealed in the cultured neurons. Additionally, animal model may have different tolerance and compensative mechanism for mitophagy impairment^49^ and ROS stress^234,235^. Target-based screening with stringent validation of mitophagy activator may help to efficiently identify promising drug candidate. The screening of mitophagy activator targeting ROS-related signalling pathways in the presence of certain antioxidant in mitophagy-intact or mitophagy-defective iPSC-derived neurons may yield compounds that potentially reverse the pathogenesis of PD. The compounds identified in mitophagy-defective neurons represent the potential drug candidates which may promote alternative mitophagy to rescue PD caused by mitophagy defects. The candidates that can promote mitophagy in the presence of antioxidant may trigger mitophagy by activating certain signalling pathway while eliminating potential ROS-related detrimental effects. With the efficient target-based screening, more efforts could be made to evaluate the effects of the hits on PD phenotypes in multiple model systems, including animal and human midbrain organoid^236^ PD model before taken into clinical trial together with the antioxidant (Fig. 3).

### Challenges and potential solutions

Therapeutic trials studying the potential of activating ROS downstream signalling pathway(s) to boost mitophagy face some challenges (Table 2). First, it is important to pinpoint mitophagy defects in relevant biological samples. In vitro culture of DA neurons or midbrain organoid derived from patient iPSCs may provide a useful tool to study mitophagy in vitro with the aid of constantly developing fluorescence dyes to readily detect mitophagy status or single cells sequencing to identify the cell populations and signalling pathways implicated in the mitophagy. Furthermore, it is of note that pioglitazone failed to halt PD progression in trials, but lowered the risk of PD, suggesting that mitophagy activation in rescuing neurodegeneration is more likely to be useful in early stages of neurodegeneration, as opposed to clinically diagnosed PD patients whereby numerous cascades of cellular dysfunctions rendering neurodegeneration are inevitable and irreversible. Thus, mitophagy activation may be more effective in the preclinical/prodromal state. Hence clinical trials should focus on asymptomatic genetic carriers or those at risk or those with prodromic symptoms. A comprehensive analysis of genetic background (known genes related to PD) and longitudinal clinical studies of blood, urine and CSF biomarkers and clinical manifestation (including symptoms of anosmia, REM disorder) will help to stratify clinical trial subjects and to monitor their progression.

**Table 2:** Key challenges and potential solution of mitophagy in future clinical research/application.

Key challenges	Potential solution
Accessibility of biological/clinical samples to assess mitophagy	DA neurons derived from patient iPSCs
Methods to assess mitophagy	Single cell RNA sequencing of the substantia nigra autopsy sample or midbrain organoid could potentially pinpoint mitophagy-deficient cells and identify druggable molecular targets. Fluorescence protein probes, such as mKeima, have been used in patient iPSC-derived neurons. Small molecule dyes, such as Mtphagy^237^, may be potentially used in patient samples.
Evaluation of stimulating ROS-related pathway(s) to promote mitophagy in PD treatment	Clinical trials to be done in prodromal patients

In conclusion, the dynamic and complex interplay between mitophagy and excessive ROS plays an important pathophysiologic role in both sporadic and familial PD. Identifying the relationship between these processes and their triggers early in the course of neurodegeneration will provide novel mechanistic clues that can potentially lead to the development of drugs that target specific pathways in this network. Proper selection of specific subsets of subjects for longitudinal clinical trials will enhance the chance of a favourable therapeutic outcome.

## Data Availability

Data sharing not applicable to this article as no datasets were generated or analysed during the current study
